# Heparin protocols in cardiovascular surgery for antiphospholipid syndrome: a clinical case report

**DOI:** 10.1186/s43044-026-00731-w

**Published:** 2026-03-30

**Authors:** Tomohiro Nakajima, Yutaka Iba, Tsuyoshi Shibata, Shuhei Miura, Takeko Hasegawa, Hidemichi Kouzu, Nobuyoshi Kawaharada

**Affiliations:** Sapporo Medical University, Sapporo, Japan

**Keywords:** Antiphospholipid syndrome, Cardiovascular surgery, Heparin

## Abstract

**Background:**

Antiphospholipid syndrome (APS) is a prothrombotic disorder that presents significant challenges during cardiovascular surgery due to its high risk of both thrombotic and hemorrhagic complications. Perioperative management of anticoagulation, particularly with heparin, requires careful balancing to prevent catastrophic outcomes such as thromboembolism or excessive bleeding.

**Case presentation:**

The patient was a 66-year-old man. Two years prior, he was diagnosed with deep vein thrombosis (DVT), and direct oral anticoagulant (DOAC) therapy was initiated. Further evaluation in the cardiology department revealed elevated anticardiolipin antibody levels, leading to a diagnosis of APS. While on DOAC therapy, he was found to have severe aortic valve stenosis caused by a bicuspid aortic valve and 45-mm dilation of the ascending aorta. Surgical intervention was deemed necessary, and the operation was performed. As activated clotting time (ACT) tends to be elevated in APS, intraoperative heparin concentration was closely monitored. The surgery proceeded without perioperative embolic events, and the patient had an uneventful postoperative course.

**Conclusions:**

We report a case of open-heart surgery in a patient with APS, where perioperative embolic events were successfully avoided through close monitoring of blood heparin concentrations.

## Background

Antiphospholipid syndrome (APS) is a prothrombotic disorder characterized by the presence of antiphospholipid antibodies, which can interfere with coagulation pathways. During open-heart surgery, activated clotting time (ACT) is routinely used to monitor anticoagulation; however, patients with APS often exhibit prolonged ACT due to the interaction of antiphospholipid antibodies with phospholipid-dependent assays. This prolongation complicates intraoperative anticoagulation management, as standard heparin dosing and ACT monitoring may not accurately reflect the anticoagulant effect, thereby increasing the risk of thrombotic or bleeding complications. Developing effective strategies for intraoperative anticoagulation monitoring and management in patients with APS undergoing cardiovascular surgery remains a significant challenge.

## Case presentation

**Fig. 1:**
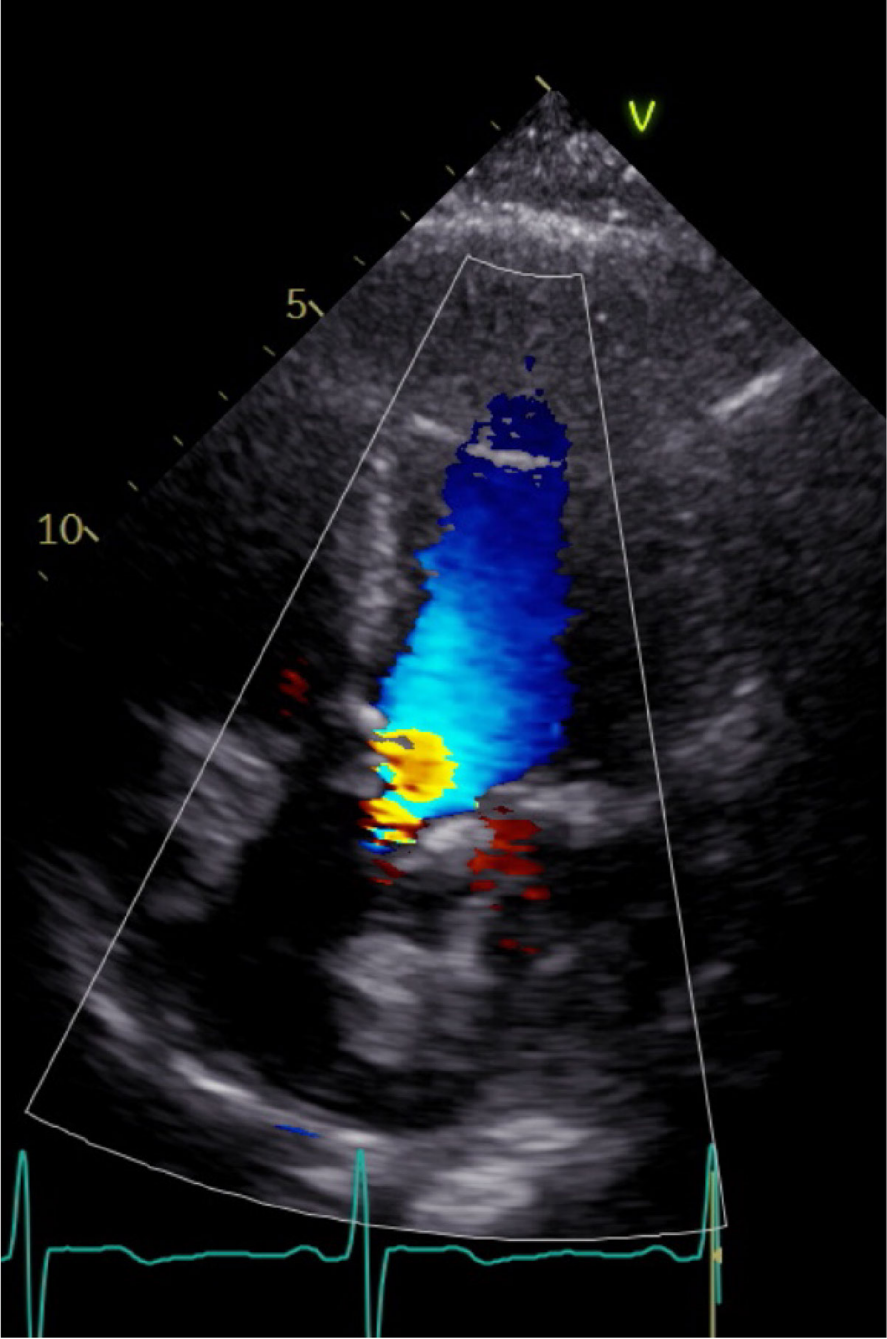
Preoperative echocardiography. Apical long-axis view showing mosaic flow and accelerated flow across the aortic valve

**Fig. 2:**
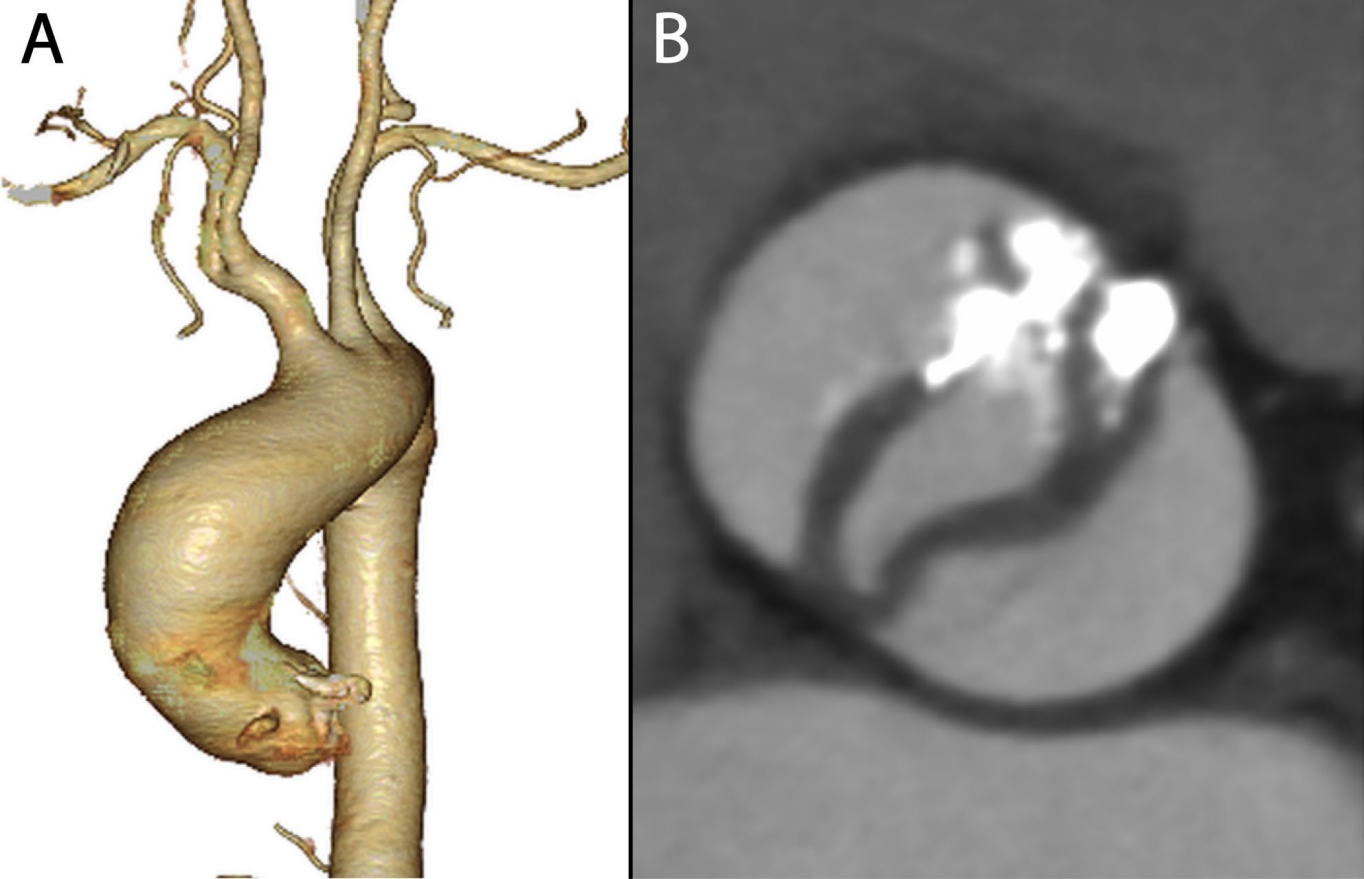
Computed tomography imaging before the operation. **A** Volume rendering image showing dilation of the ascending aorta. **B** Maximum intensity projection (MIP) image of the aortic valve demonstrating fusion of the non-coronary cusp and right coronary cusp, along with significant calcification at the commissure between the right coronary cusp and left coronary cusp

The patient was a 66-year-old man. Two years prior, he was diagnosed with deep vein thrombosis (DVT) and started on direct oral anticoagulants (DOAC). Subsequent evaluation revealed elevated anticardiolipin antibody levels, leading to a diagnosis of antiphospholipid syndrome (APS). Prior to the diagnosis of APS by a cardiologist, DOAC was prescribed instead of warfarin. The patient was monitored while continuing DOAC therapy. He later presented with exertional dyspnea, prompting further investigation. Echocardiography revealed severe aortic valve stenosis due to a bicuspid aortic valve, with an aortic valve area of 0.60 cm² (Fig. 1). Contrast-enhanced computed tomography (CT) showed a 45-mm dilation of the ascending aorta (Fig. 2). Surgical intervention was deemed necessary, and the patient underwent surgery.

Given the potential for elevated ACT values in APS, intraoperative heparin concentration monitoring was planned. The Hemochron Signature Elite^®^ system (Accriva Diagnostics, San Diego, CA, USA), Actalyke^®^ system (Helena Laboratories, Beaumont, TX, USA), and HMS Plus^®^ system (Medtronic, Minneapolis, MN, USA) were used in this operation. all of the equipment used in this case was kaolin-based. Under general anesthesia, the surgery began with a median sternotomy. After systemic heparinization, cardiopulmonary bypass (CPB) was established using a 24Fr arterial cannula in the ascending aorta and 26Fr and 30Fr venous cannulas in the superior and inferior vena cava, respectively. Heparin concentration and ACT were measured every 30 min. The ascending aorta was clamped, and cardiac arrest was induced. Myocardial protection was achieved with continuous retrograde coronary perfusion. Inspection of the ascending aorta confirmed a bicuspid aortic valve. The valve leaflets were excised, and annular calcifications were meticulously removed.

With rectal temperature lowered to 28 °C, circulatory arrest was initiated, and selective cerebral perfusion was established. We inserted a 14Fr cannula into the brachial artery, a 12Fr cannula into the left common carotid artery, and a 12Fr cannula into the left subclavian artery, and are perfusing a total flow of approximately 800 mL using a roller pump. A 26-mm J-graft prosthetic aorta was anastomosed to the distal ascending aorta using 10-mm pledgeted felt strips and continuous 4 − 0 Prolene sutures. CPB was then resumed, and rewarming commenced. Fifteen pledgeted sutures were placed in a supra-annular position, and a 25-mm Epic bioprosthetic valve was secured to the annulus. The proximal anastomosis of the graft was performed with 10-mm pledgeted felt strips and 4 − 0 Prolene sutures in a continuous fashion. The aortic cross-clamp was released.

**Fig. 3:**
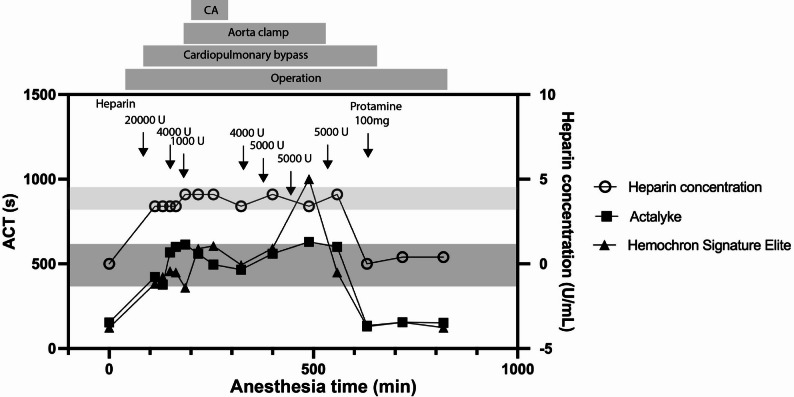
Intraoperative heparin concentration and ACT trends. The dark gray shaded area indicates the optimal ACT range during cardiopulmonary bypass (400–600 s), and the light gray shaded area indicates the optimal heparin concentration range (3.5–4.0 U/mL). *CA* circulatory arrest time

Intraoperative transesophageal echocardiography (TEE) revealed perivalvular leakage, necessitating re-clamping and cardiac arrest. Two mattress sutures were placed externally at the noncoronary cusp annulus. Upon reperfusion, trivial leakage was confirmed via TEE. CPB was discontinued, hemostasis was achieved, and the surgery concluded. Significant bleeding challenges were encountered. Hemostasis was extremely poor from the needle puncture and adipose tissue. Hemostatic agents and blood products were used to achieve hemostasis. Total circulatory arrest time was 28 min, CPB time was 130 min, aortic cross-clamp time was 157 min, and the total surgical time was 566 min. Figure 3 illustrates the intraoperative heparin concentration and ACT control.

Postoperatively, the patient was transitioned to warfarin therapy, maintaining a target PT-INR of approximately 2.0. He experienced no perioperative thromboembolic events. Follow-up echocardiography showed trivial transvalvular leakage, and contrast-enhanced CT confirmed no issues with the ascending aortic graft. The patient was discharged on postoperative day 19 in stable condition. Currently, three months of warfarin treatment for the biological valve has been completed, and treatment with a single DOAC is being administered based on the judgment of a cardiologist.

## Discussion

APS poses significant challenges during cardiovascular surgery, particularly in the monitoring of anticoagulation [1]. ACT, a standard measure during CPB, is often unreliable in patients with APS due to the interference of antiphospholipid antibodies with phospholipid-dependent coagulation assays [2]. This can lead to difficulties in assessing the true anticoagulant effect of heparin, increasing the risk of thrombotic or hemorrhagic complications. These limitations necessitate alternative or supplemental monitoring strategies to ensure optimal anticoagulation management in patients with APS undergoing open-heart surgery [3].

In this case, we prepared for surgery with two ACT measurement devices and an additional device to monitor blood heparin concentration. This strategy allowed us to cross-reference results and mitigate potential inaccuracies in ACT measurements caused by APS. By incorporating heparin concentration monitoring, we aimed to maintain a stable anticoagulant effect and ensure the safety of the surgical procedure [4, 5].

During the surgery, there was no significant prolongation of ACT. However, 500 min after the initiation of anesthesia, an abnormal ACT prolongation was observed using the Hemochron Signature Elite device. Despite this, heparin concentration remained stable, within the range of 3.5–4.0 U/mL, ensuring adequate anticoagulation [6]. The patient experienced no perioperative embolic events, and neither ACT nor heparin concentration showed significant fluctuations during deep hypothermic circulatory arrest. This highlighted the value of heparin concentration monitoring in complementing ACT, especially in cases where ACT may become unreliable [7]. In the patient with antiphospholipid syndrome (APS), using multiple ACT measurement devices with different reagent substrates (kaolin and cerite) enables comprehensive evaluation of coagulation status. Considering the variability often observed in APS patients, inter-device cross-validation improves the reliability of anticoagulation monitoring during cardiopulmonary bypass and contributes to safer surgical management. As a policy of our department, we perform circulatory arrest as a basic procedure for ascending replacement in order to perform anastomosis as close to the peripheral side as possible. The reason for this is to prevent problems caused by residual ascending aorta.

Postoperatively, unfractionated heparin was continued until the patient’s INR reached therapeutic levels with warfarin. In patients with APS, prolonged aPTT due to antiphospholipid antibodies can complicate heparin monitoring, requiring careful titration and close observation [8]. In this case, meticulous management avoided thrombotic or hemorrhagic complications, supporting the efficacy of this approach in patients with APS undergoing cardiovascular surgery.

## Conclusions

We performed aortic valve replacement and ascending aortic graft replacement in a 66-year-old patient with APS. During the surgery, prolonged ACT values were observed with the ACT monitoring device due to the effects of heparin administration. However, by concurrently monitoring heparin concentration, we were able to maintain stable anticoagulation and safely proceed with the surgery. The patient had an uneventful perioperative course, with no thromboembolic complications observed. This case highlights the importance of combining ACT and heparin concentration monitoring to ensure effective anticoagulation management in patients with APS undergoing cardiovascular surgery.

## Data Availability

No datasets were generated or analysed during the current study.

## Abbreviations

APS: Antiphospholipid syndrome
ACT: Activated clotting time
